# Unusual phylogenetic tree and circulating actionable ESR1 mutations in an aggressive luminal/HER2-low breast cancer: Case report

**DOI:** 10.3389/fonc.2022.1050452

**Published:** 2023-01-11

**Authors:** Matteo Allegretti, Vittoria Barberi, Cristiana Ercolani, Antonello Vidiri, Elena Giordani, Gennaro Ciliberto, Patrizio Giacomini, Alessandra Fabi

**Affiliations:** ^1^ Translational Oncology Research, IRCSS Regina Elena National Cancer Institute, Rome, Italy; ^2^ Medical Oncology 1, IRCSS Regina Elena National Cancer Institute, Rome, Italy; ^3^ Pathology, IRCSS Regina Elena National Cancer Institute, Rome, Italy; ^4^ Radiology and Diagnostic Imaging, IRCSS Regina Elena National Cancer Institute, Rome, Italy; ^5^ Scientific Directorate, IRCSS Regina Elena National Cancer Institute, Rome, Italy; ^6^ Clinical Trial Center, IRCSS Regina Elena National Cancer Institute, Rome, Italy; ^7^ Precision Medicine in Senology, Scientific Directorate - Department of Women and Child Health, Fondazione Policlinico Universitario "A. Gemelli" IRCCS, Rome, Italy

**Keywords:** breast cancer, cancer evolution, liquid biopsy, ESR1, HER2, molecular tumor board

## Abstract

Under therapeutic pressure aggressive tumors evolve rapidly. Herein, a luminal B/HER2-low breast cancer was tracked for >3 years during a total of 6 largely unsuccessful therapy lines, from adjuvant to advanced settings. Targeted next generation sequencing (NGS) of the primary lesion, two metastases and 14 blood drawings suggested a striking, unprecedented coexistence of three evolution modes: punctuated, branched and convergent. Punctuated evolution of the trunk was supported by *en bloc* inheritance of a large set (19 distinct genes) of copy number alterations. Branched evolution was supported by the distribution of site-specific SNVs. Convergent evolution was characterized by a unique asynchronous expansion of three actionable (OncoKB level 3A) mutations at two consecutive ESR1 codons. Low or undetectable in all the sampled tumor tissues, ESR1 mutations expanded rapidly in blood during HER2/hormone double-blockade, and predicted life-threatening local progression at lung and liver metastatic foci. Dramatic clinical response to Fulvestrant (assigned off-label exclusively based on liquid biopsy) was associated with clearance of all 3 subclones and was in stark contrast to the poor therapeutic efficacy reported in large liquid biopsy-informed interventional trials. Altogether, deconvolution of the tumor phylogenetic tree, as shown herein, may help to customize treatment in breast cancers that rapidly develop refractoriness to multiple drugs.

## 1 Introduction

Hormone-receptor (HR) positive breast cancer includes the luminal A and luminal B subtypes and is the most common type of tumor in women diagnosed with early-stage breast cancer. Although its overall prognosis is better than HER2 + and triple-negative breast cancers, individual risk of relapse may differ widely (1, 2).

The mainstay of targeted treatment for luminal breast cancer is endocrine therapy (ET), which includes several agents that either directly target estrogen receptor (ER) or suppress estrogen production. Standard adjuvant treatment in low-risk breast cancer involves Tamoxifen in pre-menopausal patients, and aromatase inhibitors (Ai) in post-menopausal patients. Association of luteinizing hormone-releasing hormone inhibitors is recommended in pre-menopausal patients. Cyclin-dependent kinase inhibitors (CDK4/6i), combined with ET, are recognized as the standard of care for adjuvant treatment in high-risk patients, and in the metastatic setting (1, 2). Trastuzumab Deruxtecan (T-DXd), recently approved for HER2^+^ advanced tumors, is expected to have a strong clinical impact on HR^+^/HER2-low luminal breast cancers (3).

Despite expanding therapeutic options, some invasive luminal breast cancers may rapidly develop resistance to pharmacological treatment. In many tumors, this has been associated with biological aggressiveness and complex sub-clonal dynamics (4, 5). It is becoming increasingly clear that real-time tracking by longitudinal tumor tissue sampling and liquid biopsy (LB) would be useful to instruct clinical decisions (5) but, with notable exceptions (6), phylogenetic trees are mostly reconstructed by genome-wide *post-mortem* analysis of tumor DNA from multiple metastatic foci.

Herein, we describe an aggressive breast carcinoma characterized by the co-occurrence of an extremely fast clinical progression and an unusual molecular evolution. Whereas its luminal B/HER2-low subtype features were conventional and poorly informative, targeted next generation sequencing (NGS) of tissue and liquid biopsies suggested an unprecedented combination of punctuated, branched, and convergent evolution patterns (4). Clinical NGS deciphered tumor phylogenesis and convergent adaptive selection, enabling successful assignment of off-label treatment.

## 2 Case description

**Figure 1:**
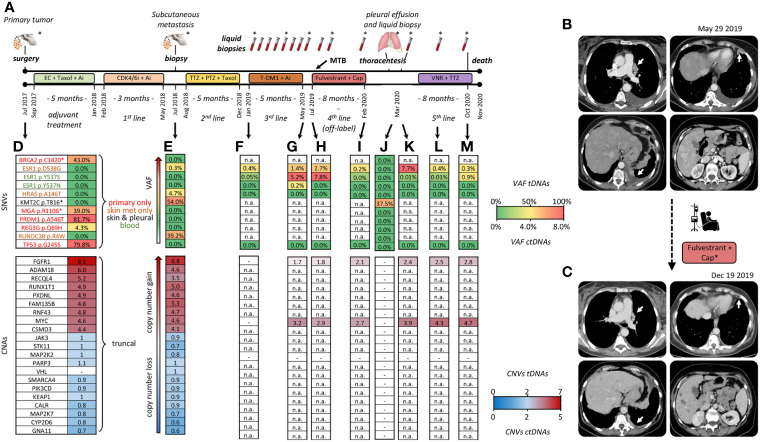
Clinical timeline and tumor profiling. **(A)** Treatment history: adjuvant chemotherapy with Epirubicine plus Cyclofosphamide (EC: 90/600 mg/mq, q14), followed by Taxol (175 mg/mq, q14) plus Pegfilgrastim, and Aromatase Inhibitor (Ai: Letrozole 2.5 mg/day). First line therapy with Palbociclib (CDK/6i:125 mg/die for 21 days, every 28 days) plus Aromatase Inhibitor (Ai: Letrozole 2.5 mg/day). Surgical removal of a thoracic skin metastasis. Second line with Trastuzumab (TTZ: 8 mg dose loading → 6 mg) plus Pertuzumab (PTZ: 840 mg dose loading → 420 mg) and weekly Taxol. Third line with T-DM1 (3.6 mg/kg, every 21 days) and Letrozole (2.5 mg/day), beginning of LB monitoring. Fourth line, off-label Fulvestrant (500 mg day 1, 15, 28, then every 28 days) in association with Capecitabine (Cap: 2000 mg/m^2^ daily discontinued after a single cycle). Fifth line with Vinorelbine (VNR: 25 mg/mq at day 1 and 8) plus Trastuzumab (8 mg/kg -> 6 mg/kg) every 21 days. Asterisks: tissue and blood samples tested by both NGS and dPCR. All the other samples: testing by dPCR only. **(B, C)** CT scans before and following treatment with Fulvestrant; dates of clinical imaging are noted. Major sites of response to Fulvestrant are indicated by white arrows. Cap*, Capecitabine **(D–M)** Key time points at which tumor tissue/cells and blood drawings were obtained. Single nucleotide variants (SNV) are color-coded by tissue of origin in **(D)**. Palettes rank alterations by variant allele frequency (different %VAFs) and copy numbers (CNA). Copy numbers are expressed per diploid genome: copy number-neutral n=2. tDNA, tumor DNA; ctDNA, circulating tumor DNA. (-) undetectable. n.a., not assessed because SNV/CNA was not included in the LB-grade NGS panel.

The clinical timeline, treatments and main diagnostic assessments in this patient are summarized in **Figure 1A**. In July 2017, a 55-year-old woman underwent quadrantectomy and axillary lymphadenectomy for a multifocal (G3) ductal infiltrating breast carcinoma of the luminal B subtype: estrogen receptor (ER) 90%, progesterone receptor (PgR) 5%, Ki67 50%, HER2-low (immunohistochemistry 2+; non-amplified by *in situ* hybridization). In December 2017, one month only after the end of adjuvant therapy, early metastatic dissemination to the lung, bones and parasternal soft tissue called for first-line therapy with CDK4/6i plus Letrozole. Unfortunately, further rapid progression occurred (July 2018) involving the lung and spine (L2/L3), which prompted to surgical removal of a thoracic subcutaneous metastasis for diagnostic re-assessment and targeted NGS (see below). Routine immunohistochemistry of the metastatic tissue confirmed the ER^+^/PgR^+^/HER2 2^+^ status detected in the primary, and in addition revealed a very slight HER2 copy number gain (HER2/CEP17 SISH ratio: 2.4). Since Trastuzumab deruxtecan (3) was not available in year 2018, a combined regimen was attempted of Trastuzumab plus Pertuzumab and weekly Taxol for double HER2/hormone blockade (2^nd^ line). However, novel pleural and left costal lesions developed after 5 cycles. This prompted us to initiate 3^rd^ line therapy with Trastuzumab emtansine (T-DM1) plus Letrozole. This combination was administered in the context of a LB study, now published (7). LB detected progressive accumulation of 3 actionable ESR1 circulating alterations (see below) during treatment, despite apparently stable disease. Clinical progression (massive dissemination to the lung, pleura and liver) occurred in June 2019 (total body CT scan, **Figure 1B**). The patient was referred to the Regina Elena intramural Molecular Tumor Board (MTB) in very poor general conditions and dyspnoic. Off-label Fulvestrant was recommended (see below) exclusively based on LB data, in association with Capecitabine (4^th^ line), but the latter had to be discontinued after a single cycle due to severe gastrointestinal intolerance. In contrast, Fulvestrant was well tolerated and, despite administration as single-agent, it resulted in a quick and dramatic improvement with disappearance of most lung lesions and associated pleural exudate, as well as drastic regression of the extensive liver invasion (**Figure 1C**). Response lasted 8 months until early February 2020, when the patient, who was otherwise in good general conditions and essentially asymptomatic, suddenly experienced dyspnea and chest pain due to a pleural exudate requiring thoracentesis. Salvage treatment with Trastuzumab plus Vinorelbine (5^th^ line) resulted in partial response of the pleural effusion and apparently stable thoracic disease for about 7 additional months, until massive pleural/lung recurrence and death in November 2020.

## 3 Diagnostic assessment and molecular profiling

Tumor DNAs (tDNAs) were obtained from the three sampled tumor lesions: the primary tumor, the subcutaneous metastasis, and neoplastic cells isolated from the pleural effusion following overnight adherence to plastic dishes. Circulating tumor DNAs (ctDNAs) were obtained from 14 blood drawings. All 3 tDNAs and 8/14 ctDNAs (**Figure 1A**, asterisks) were tested by NGS (Life Technologies, see Supplementary for technical details) with two targeted panels of different complexity. The tDNA-grade Oncomine™ Comprehensive Assay Plus detected 9 single nucleotide variants (SNVs) and 19 copy number alterations (CNA) in a total of 505 tested genes. These 27 alterations are listed along with variant allele frequencies (VAFs) and estimated copy numbers in **Figures 1D, E, J**. Due to its smaller size (it detects SNVs, CNAs and fusions in 52, 12 and 92 genes, respectively), the ctDNA-grade Oncomine™ PanCancer Cell-Free Assay could only assess 3 of 9 SNVs and 2 of 19 CNAs. Results are shown for 7 representative blood drawings (**Figures 1F–M**; n.a.: not assessed).

The distribution of the 9 SNVs among the three tDNAs readily revealed marked phylogenetic divergence during tumor evolution: 5 SNVs (BRCA2 p.C1820, MGA p.R1106*, PRDM1 p.A546T, REG3G p.Q69H and TP53 p.G245S) were exclusive of the primary, 3 (HRAS p.A146T, RUNDC3B p.R4W and ESR1 p.D538G) were exclusive of the subcutaneous metastasis, and only one (KMT2C p.T816) was shared between any two lesions, namely between the subcutaneous metastasis and the neoplastic pleural effusion (**Figures 1D, E, J**, top section; SNVs color-coded by site of detection in panel d).

Particularly in light of rapid clinical progression, BRCA p.C1820 and TP53 p.G245S losses appeared counterintuitive. The former is a potentially inheritable, likely pathogenic variant (https://varsome.com), and the latter is a loss-of-function cancer driver strongly associated with poor outcome (OncoKB level Px1; https://www.oncokb.org). These considerations prompted to orthogonal testing by custom-designed digital PCR (dPCR) assays. However, NGS calling was confirmed, and dPCR-assessed VAFs differed marginally (within +/- 3%) from NGS-assessed VAFs (see representative TP53 testing in **Table S1**). Likewise, dPCR confirmed that BRCA2 p.C1820 was undetectable at both tested metastatic sites and in leukocyte DNA (not shown), conclusively ruling out a germ line origin.

Next, we focused on the similarly puzzling observation of a simultaneous presence of three gain-of-function ESR1 SNVs (p.D538G, p.Y537S and p.Y537N), all known to be actionable and associated with resistance to hormone blockade (8). Interestingly, these three SNVs displayed very different origins and accumulation kinetics, as may be seen by comparing tDNA from the skin metastasis, and ctDNAs obtained before the T-DM1/Letrozole and Fulvestrant treatments, at progression, and at later time points (**Figures 1E–M**). Specifically, ESR1 p.D538G was the only ESR1 alteration to be detected in tissues. It displayed a very low VAF at onset both in tDNA and ctDNA, and then marked blood increases mostly coincident with clinical progression. In contrast, ESR1 p.Y537S and p.Y537N, initially undetectable, appeared *de novo* in blood, and their association with clinical outcome appeared to be less stringent. None of the three SNVs could be detected in the pleural effusion (**Figure 1J**). Also these changes were confirmed by orthogonal dPCR validation (representative results shown in **Table S1**). In summary, BRCA2 p.C1820 and TP53 p.G245S were negatively selected, whereas ESR1 alterations were positively selected by successive events taking place for the most part at undetermined tumor sites.

Strikingly in contrast to the heterogeneous SNV distribution, the primary site and the skin lesion did share a large set of 19 collinear CNAs (**Figure 1D, E**, bottom section). Most CNAs appeared to be inherited *en bloc*, since copy number gains and gene-to-gene ratios were roughly conserved between the two lesions. Only FAM135B displayed a discordant trend (increase instead of decrease/stability in copy numbers). Unfortunately, not all CNAs were testable by the ctDNA-grade NGS panel. Despite this limitation, we were able to confirm that both FGFR1 and MYC (the only two CNAs testable in blood) were amplified and, similar to tissues, copy number gains persisted in serial blood drawings, undergoing limited absolute and relative variations (**Figures 1F–L**, bottom). Of special interest, FGFR1 and MYC amplifications were detected in peripheral blood collected at the time of thoracentesis despite tDNA from the pleural neoplastic cells had lost FGFR1, MYC, and all the other CNAs (**Figure 1J vs K**). The easiest interpretation of these results is that a clonal expansion containing at least FGFR1 and MYC CNAs was present throughout disease course, e.g. in the primary tumor, skin metastatic site and other unidentified tumor sites, as indirectly revealed by LB, but it was lost in the last lesion that had been sampled, e.g. tumor cells from the pleural cavity.

**Figure 2:**
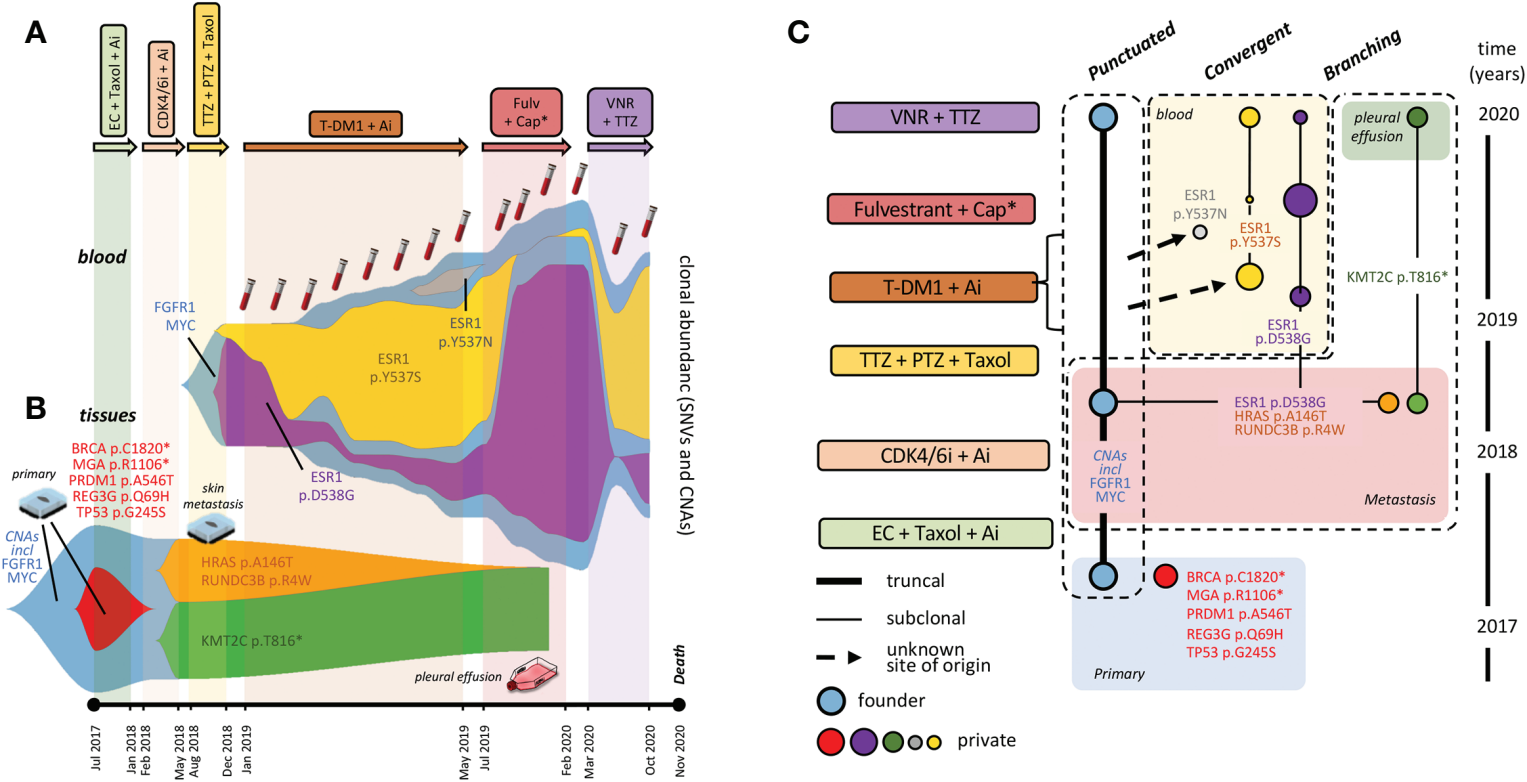
Fishplots and tumor phylogenesis. Fishplots displaying alterations in blood **(A)** and tumor lesions **(B)**. Areas are proportional to relative SNV abundances, whereas CNAs are depicted in background, not to scale. **(C)** Tumor phylogenetic tree: boxes surround evolution modes. Connecting lines: parental relationship proven (continuous) and inferred (dotted). ESR1 mutations: dimension of dots (blood variants) is roughly proportional to clonal representation. Tumor clones are consistently color-coded between fishplot areas and the dots of the phylogenetic tree. Abbreviations as in **Figure 1**.

To display multiple SNV and CNA trajectories, longitudinal series of %VAF values and DNA copy numbers were elaborated by the fishplot package for RStudio (9). The 6 ctDNAs not tested by NGS were included by incorporating dPCR data in the model. To portray tumor evolution in its entirety we chose to represent SNVs and CNAs in the same graph, and generated separate fishplots for blood and tumor lesions (**Figures 2A, B**). For simplified CNA rendering, we focussed on FGFR1 and MYC copy numbers, that are available for both blood and tissues. These were averaged and merged into a single fishplot. However, since %VAF and gene copy numbers are incommensurable, the latter were displayed in background and not to scale. It is acknowledged that no inference can be made about relative SNV/CNA subclonal representation, whereas quantitative relationships among multiple SNVs are faithfully graphed.

Specifically, the fishplot in **Figure 2A** clearly shows relative abundance and kinetics of ESR1 p.D538G p.Y537S and p.Y537N. Quite strikingly, the two most abundant variants displayed a roughly reciprocal relationship whereby expansion of either is mirrored by contraction of the other. Unsurprisingly, circulating ESR1 alterations were prodromic to life-threatening clinical progression (see case description), which led to seek MTB multidisciplinary advice.

In elaborating therapeutic recommendations, the MTB considered the following points: (a) the 3 actionable (OncoKB level 3A) ESR1 SNVs had disabled two consecutive codons in the catalytic ESR1 site; (b) they had occurred in rapid succession within 7 months from the first T-DM1/Letrozole administration; (c) their distinct kinetics and tissue/blood distribution were suggestive of convergent adaptive events in distinct founders; (d) their timing of appearance and subsequent clinical progression at lung and liver foci was compatible with an elective topographical distribution of at least some ESR1 variants at these sites.

Preliminary evidence was also considered from the plasmaMATCH trial, now published (10), that subclonal ESR1 alterations when detected in blood do not confer sensitivity to targeted treatment. Nevertheless, in light of arguments (a-d), and in the absence of alternative therapeutic options, off-label Fulvestrant was recommended exclusively based on LB data, and in association with Capecitabine.

As outlined in case description, only Fulvestrant was tolerated, and dramatic clinical improvement (CT scans in **Figure 1C**, performed in November) was mirrored, in December 2019, by nearly complete blood clearance of the newly arisen ESR1 p.Y537S and p.Y537N variants, although the ESR1 p.D538G clone (chronologically the first to appear) re-expanded, and FGFR1/MYC CNAs kept expanding steadily in blood (**Figure 2A**). These results formally prove direct variant (particularly ESR1 p.Y537S) targeting by Fulvestrant, and provide evidence for a dissociated response, both molecular and clinical.

At the time of further pleural relapse in early February 2020, the ESR1 p.D538G variant and the FGFR1 and MYC copy number gains were all abundant in blood (**Figure 2A**). However, none was detectable in tumor cells obtained on the same day from the neoplastic pleural effusion, that only retained a single ‘undruggable’ KMT2C SNV (**Figure 2B**) first seen in the 2018 cutaneous metastasis. While further supporting clearance of ESR1 variants from the pleural space, these results prevented target FGFR1 treatment, and prompted instead to Trastuzumab plus Vinorelbine as a possible salvage therapy. Partial response of the pleural effusion was accompanied by decreases in FGFR1, MYC and ESR1 p.D538G variants, and (re)-expansion of ESR1 p.Y537S (**Figure 2A**). Subsequent increases of all variants in October 2020 (**Figure 2A**, last blood drawing) heralded massive pleural/lung recurrence and death in November 2020.

For synoptic view, the CNA trunk and the 11 SNVs detected in tDNAs and ctDNAs were assigned to specific tumor lesions and blood drawings by applying the pigeonhole principle and logical deduction. The inferred tumor phylogenetic tree is shown in **Figure 2C**.

## 4 Discussion

Herein, we describe an aggressive HER2-low breast cancer that, based on straightforward immunohistochemical and molecular subtyping, apparently meets the ‘standard’ definition of a luminal B tumor subtype. However, extensive NGS profiling clearly shows that its hidden hallmark is the unprecedented (to our knowledge) co-existence of punctuated, branched, and convergent evolution (boxed in **Figure 2C**).

Punctuated evolution is supported by persistence of a large set of collinear CNAs (particularly the FGFR1/MYC amplifications) across the primary, the skin metastasis, and possibly other tumor sites (as indirectly supported by LB). *En bloc* inheritance of multiple alterations in the tumor trunk is a hallmark of punctuated evolution (5).

Convergent evolution of the three ESR1 subclones is supported by their distinct origin and timing of appearance (e.g. one from the skin metastasis and two from unknown metastatic sites), and their striking convergence toward the same phenotypic effect (resistance to hormone blockade).

Finally, branched evolution is best exemplified by the localization of the ‘undruggable’ KMT2C variant (likely originated from the skin lesion) to the second neoplastic pleural effusion in the absence of all other alterations, including actionable FGFR1 amplification and ESR1 mutations. This is compatible with a dramatic clonal sweep. This event had implications on therapeutic escape, as it left no residual targeting options.

Single-cell omics of tumor tissues and circulating tumor cells have shown that each breast cancer is a collection of multiple molecular subtypes, and may be viewed as a dynamically evolving, adaptively selected cell ecotype (11, 12). It is tempting to speculate that multi-modal evolution, detected herein, is a powerful shortcut to ecotype plasticity and tumor aggressiveness.

The closest precedent of the present study is possibly a case report in which treatment-induced regression of actionable genomic alterations at specific metastatic sites correlated with changes in the corresponding ctDNAs (13). However, in this previous report the tumor architecture was reconstructed *post-mortem*, whereas in the present study the entire deduction process was in real-time and based on *ex vivo* testing. Consequently, NGS/dPCR results could be exploited for therapeutic purposes.

Response of ESR1-mutated variants (OncoKB level 3A when detected in tissues) to Fulvestrant deserves a comment in light of poor success (0/27 objective responses when subclonal ESR1 alterations were detected in blood) of the PlasmaMATCH trial (10). At least four arguments support the idea that the single patient described herein is an exceptional responder: (a) an unusual oncogenic dependence from ESR1, supported by convergent evolution of three variants; (b) selective metastatic seeding at an anatomical site (lung) electively responsible for life-threatening progression in this specific patient; (c) reciprocal expansion/contraction of ESR1 subclones during therapy, e.g. clonal competition for the same ecotype niche, a condition suggested, but not proven, to result in self-limited tumor spreading (4); and (d) Fulvestrant-mediated simplification of the tumor phylogenetic tree, that may have helped to also achieve partial response to subsequent salvage therapy of the undruggable KT2MC-associated pleural variant.

One may object that the benefit of single-agent treatment with Fulvestrant, although impressive, was rather short-lived. Based on the I-PREDICT drug combination trial (14) we hypothesize that a double FGFR1/ESR1 blockade acting on both the trunk and the first lung/liver tumor branch(es) might have been better to counteract multiple evolution modes, resulting in a more durable response. At any rate, the patient survived 18 months after progression from the last conventional therapeutic option, which is remarkable in light of the short overall survival time (about 40 months) from diagnosis to death.

Tumors with no special or notable features except inexplicable clinical aggressiveness may not be unusual in the clinical practice. Our study suggests that deconvoluting the tumor phylogenetic tree by clinical-grade NGS may suffice to customize treatment in breast cancers that rapidly develop refractoriness to multiple drugs.

## 5 Patient perspective

The patient was a business manager with key professional responsibilities in her company. During the entire clinical course she was fully aware of her clinical conditions, and specifically asked the medical oncologist to receive all the necessary treatments (including off-label Fulvestrant), as long as she could stay sufficiently fit to attend her duties.

## Data availability statement

The datasets presented in this study can be found in online repositories. The names of the repository/repositories and accession number(s) can be found in the article/**Supplementary Material**.

## Ethics statement

The studies involving human participants were reviewed and approved by Fondazione Ugo Bietti, *via* Elio Chianesi 53, Rome, Italy. The patients/participants provided their written informed consent to participate in this study.

## Author contributions

VB recruited and monitored the patient, MA performed and analysed NGS and dPCR. CE and EG processed tissue and plasma specimens. AV reviewed CT scans. GC supervised and coordinated MTB discussions. MA, PG, and AF planned study activities and wrote the paper. All authors contributed to the article and approved the submitted version.
