# Supplementary material for: Unusual phylogenetic tree and circulating actionable ESR1 mutations in an aggressive luminal/HER2-low breast cancer: Case report
Source: Front Oncol. 2023 Jan 11;12:1050452. doi: 10.3389/fonc.2022.1050452 (PMC9874630; doi:10.3389/fonc.2022.1050452)
Supplement: Supplementary file 1 [file DataSheet_1.pdf]

Supplementary Material

Unusual phylogenetic tree and circulating actionable ESR1 mutations in an aggressive luminal/HER2-low breast cancer: case report

1 Supplementary Data

Clinical specimens. Tumor tissues and blood drawings were collected, processed and biobanked as per international good laboratory practice. Briefly, sections (5 µm-thick) were cut from a representative formalin-fixed, paraffin-embedded (FFPE) tissue block or core biopsy from primary and metastatic lesions. One section was counterstained by hematoxylin/eosin and assessed for quality by an expert pathologist. The others were processed by the QIAmp DNA/RNA FFPE Tissue Kit (Qiagen) according to the manufacturer's instructions. Whole blood (30ml) was drawn in BD Vacutainer K2EDTA tubes, and processed within 30 min from blood drawing. Plasma was isolated by two successive rounds of centrifugation at 4°C (2000 x g for 20 min, and 13000 x g for 10 min), and stored at -80° in single-use 2 ml aliquots until extraction of circulating free nucleic acids (DNA and RNA). No freeze-thawing cycles were allowed. Circulating nucleic acids were extracted from 4 ml of plasma by the QIAmp Circulating Nucleic Acid kit (Qiagen) according to the manufacturer's instructions, and stored at -20°C until analysis. Genomic DNA from matched plasma-depleted whole blood (e.g leukocytes) was extracted by the DNeasy Blood and Tissue kit (Qiagen). Nucleic acids were fluorimetrically quantified with the Qubit dsDNA HS assay kit (Life Technologies, Carlsbad, CA, USA).

NGS. Tissue NGS libraries were prepared from 40 ng of DNA/RNA with the Oncomine Comprehensive Assay Plus (Life Technologies), as per manufacturer's instructions. This panel covers over 500 unique genes, enabling the detection of single gene SNVs, indels, CNAs, known and novel fusions, and splice variants. For blood NGS analysis, 40 ng of circulating nucleic acids were used as input to prepare libraries with the Oncomine™ PanCancer Cell-Free Assay (Life Technologies). This panel generates an amplicon library covering 52 genes, 12 copy number variations (CNAs) and 92 fusions. Libraries were equalized, pooled and then automatically loaded onto the Ion 550 (tissue) or 540 chips (blood) in the Ion Chef system (Life Technologies). After sequencing on Ion S5, data were analyzed with the Ion Reporter suite v5.18 (Life Technologies). The preset Oncomine Extended and Oncomine Variants filters v5.18 (Life Technologies) were used to call alterations in tissue and blood samples, respectively. Raw NGS and dPCR data are available from the corresponding author on reasonable request.

dPCR. Primers and probes were designed with the Custom Taqman® Assay Design Tool (CADT, Life Technologies). Matched tissue and blood samples from patients and blood from healthy donors were run in the same experiment using the chip-based QuantStudio™ 3D Digital PCR System (Life Technologies). Reactions were set up in a final volume of 16 µl including 8 µl of 2x Master Mix, 0.9 nM of each forward and reverse primers, 0.25 nM of TaqMan® MGB probe, 7.0 µl of template, and loaded onto dPCR chips. Input DNA for tissue analysis was normalized to 20 ng. By contrast, input cfDNA was equalized by volume (7.2 µl) to accurately measure ctDNA copies/mL of plasma. Thermal cycling was as follows: 10 min at 96.0°C, 39 cycles at 56.0°C for 2 min, 30 sec at 98.0°C, and a final elongation step of 2 min at 60°C. Threshold values of FAM and VIC fluorescence were automatically

calculated by the Thermo Fisher Cloud Analysis Suite, manually reviewed, and then applied to the corresponding tissue or blood sample.

Table S1

Orthogonal testing by NGS and dPCR.

		NGS VAF*	dPCR VAF
Primary lesion (2017)	TP53 p.G245S	84,4%	82,0%
	ESR1 p.D538G	0,0%	0,0%
	ESR1 p.Y537S	0,0%	0,0%

Skin metastasis (2018)	TP53 p.G245S	0,0%	0,0%
	ESR1 p.D538G	0,3%	0,3%
	ESR1 p.Y537S	0,0%	0,0%

*variant allelic frequency
